# Body sway predicts romantic interest in speed dating

**DOI:** 10.1093/scan/nsaa093

**Published:** 2020-07-18

**Authors:** Andrew Chang, Haley E Kragness, Wei Tsou, Dan J Bosnyak, Anja Thiede, Laurel J Trainor

**Affiliations:** Department of Psychology, Neuroscience and Behaviour, McMaster University, Hamilton L8S 4K1, Canada; Department of Psychology, Neuroscience and Behaviour, McMaster University, Hamilton L8S 4K1, Canada; Department of Psychology, Neuroscience and Behaviour, McMaster University, Hamilton L8S 4K1, Canada; Department of Psychology, Neuroscience and Behaviour, McMaster University, Hamilton L8S 4K1, Canada; McMaster Institute for Music and the Mind, McMaster University, Hamilton L8S 4K1, Canada; Department of Psychology, Neuroscience and Behaviour, McMaster University, Hamilton L8S 4K1, Canada; Cognitive Brain Research Unit, Department of Psychology and Logopedics, Faculty of Medicine, University of Helsinki, Helsinki 00014, Finland; Department of Psychology, Neuroscience and Behaviour, McMaster University, Hamilton L8S 4K1, Canada; McMaster Institute for Music and the Mind, McMaster University, Hamilton L8S 4K1, Canada; Rotman Research Institute, Baycrest Hospital, Toronto M6A 2E1, Canada

**Keywords:** interpersonal interaction, romantic interest, groove, Granger causality, mixed effect model

## Abstract

Social bonding is fundamental to human society, and romantic interest involves an important type of bonding. Speed dating research paradigms offer both high external validity and experimental control for studying romantic interest in real-world settings. While previous studies focused on the effect of social and personality factors on romantic interest, the role of non-verbal interaction has been little studied in initial romantic interest, despite being commonly viewed as a crucial factor. The present study investigated whether romantic interest can be predicted by non-verbal dyadic interactive body sway, and enhanced by movement-promoting (‘groovy’) background music. Participants’ body sway trajectories were recorded during speed dating. Directional (predictive) body sway coupling, but not body sway similarity, predicted interest in a long-term relationship above and beyond rated physical attractiveness. In addition, presence of groovy background music promoted interest in meeting a dating partner again. Overall, we demonstrate that romantic interest is reflected by non-verbal body sway in dyads in a real-world dating setting. This novel approach could potentially be applied to investigate non-verbal aspects of social bonding in other dynamic interpersonal interactions such as between infants and parents and in non-verbal populations including those with communication disorders.

## Introduction

Romantic interest is one of the most essential forms of social bonding and is fundamental to human society. Many previous studies have investigated social and personality factors related to the formation, maintenance, happiness and outcome of romantic relationships (e.g. Gottman & Gottman, 2017; Gottman & Levenson, 2000), but few have examined non-verbal aspects of initial romantic interest or the potential role of background music in its formation.

Initial romantic interest in real-world settings can be examined using speed dating, a matchmaking process in which people have a series of short dates with potential romantic partners, because it simultaneously allows experimental manipulation and high external validity (see Finkel and Eastwick, 2008 for a review). The speed dating context has been widely used to investigate social and personality factors related to romantic interest (e.g. Joel *et al.*, 2017). However, the role of interpersonal interaction and non-verbal behavior in initial romantic interest has rarely been studied scientifically, despite being viewed as a crucial factor by the general public (e.g. Reiman, 2012). Here, we measure how the interactive body sways of dyad members engaged in speed dating relate to romantic interest.

Body sway is a non-verbal behavioral index reflecting real-time interpersonal interactions across many settings. People rarely sit or stand perfectly still, but rather engage in subtle sway of their torso and head back and forth as a unit, typically without awareness. When people coordinate joint actions, have a conversation, have a psychotherapy session or play music in an ensemble, their body sways tend to be coupled (e.g. Goebl and Palmer, 2009; Shockley *et al.*, 2009; Ramseyer and Tschacher, 2011; Pezzulo *et al.*, 2019). Although hand gestures and other types of movement can play a role in interpersonal interactions, body sway is a global measurement of an individual’s actions that continues over time; furthermore, it is less affected by task-specific movement requirements than, for example, hand or arm gestures (Shockley *et al.*, 2009).

For each speed date, we examined the coupling relationships between the two time series representing the body sways of each partner across the 4 min of the date. We examined both directional and similarity couplings between their body sways. Directional coupling was indexed by Granger causality (GC). It determines to what extent the movements of one partner at each point in time can be predicted, or even influenced, by how the other partner just moved. It is calculated after controlling for predictions within each time series, that is, how well a person’s movements can be predicted from their own past movements. It is a directional measure in that the coupling strength (i.e. predictive power) of each partner on the other can be separately calculated, and it is possible that one partner’s body sway can better predict the body sway of the other compared to vice versa. Such directional coupling dynamics have been shown to relate to social outcomes. For example, GC analyses of body sway among musicians have shown that body sway couplings reflect leader–follower relationships (leaders predict followers more than vice versa), the quality of joint performances (higher-rated performances have higher coupling strength) and aesthetic emotional expression (expressive performances have higher coupling strength than non-expressive performances) (Chang *et al.*, 2017, 2019).

At the same time, a parallel body of work has shown that interpersonally synchronized or similar movements, which can be indexed by cross-correlational (CC) coupling, are associated with interpersonal affiliation and cooperation (e.g. Hove and Risen, 2009; Wiltermuth and Heath, 2009; Tarr *et al.*, 2015; Trainor and Cirelli, 2015; Cirelli, 2018). It is important to distinguish these two measures of coordination as they relate to interpersonal movement. CC quantifies the similarity between two time series, and GC quantifies the directional predictive power of each time series on the other. Thus, two people swaying precisely in time together would have a high CC value but low GC values, because one partner’s past movements cannot help predict the other partner’s current movements over and above prediction by that person’s own past movements if they are the same as those of their partner. On the other hand, even if two people sway very differently and therefore have a low CC value, they can still have a high GC value in one or both directions, as long as there is a robust predictive relationship between their body sways. Here, we investigated whether directional (GC) and/or similarity (CC) interpersonal body sway coupling during speed dating reflect romantic interest.

Dating environments typically include background music; however, the effect of music on romantic interest has rarely been studied. Indeed, many settings where people experience romantic interactions, such as restaurants, bars, parties and dances, have music. Music is known to drive both intentional movements and those occurring outside of awareness, especially in the context of styles common in soul, funk and jazz that are known to be high in ‘groove.’ Groove is defined as the extent to which the music makes one want to move (Janata *et al.*, 2012). Empirical studies have shown that high groove music promotes entrainment of body sway to the beat of the music (e.g. Ross *et al.*, 2016). Therefore, we further hypothesized that groove might promote romantic interest by enhancing interpersonal body sway coupling.

To investigate these hypotheses, we set up a real speed dating event. After each 4 min date, participants recorded their romantic interest in their partner (interest in meeting again, interest in pursuing a short- and a long-term relationship) and their partner’s attractiveness. Body sway during each date was recorded while high- or low-groove music was played in the background. At the end of the event, contact information was given to those who matched.

## Methods

### Participants

Fifty-five participants attended one of two speed dating sessions. Fourteen men (age 27.9 ± 2.3, range 25–32 years) and 14 women (age 29.6 ± 4.3, range 25–36 years) attended the first session (one woman’s data were excluded from analyses due to missing responses). Thirteen men (age 28.8 ± 2.8, range 25–34 years) and 14 women (age 27.1 ± 2.3, range 25–32 years) participated in the second session. The recruitment criteria included: aged 25–35 years, single, interested in a relationship with the opposite gender and fluent in English. Participants were recruited within the city of Hamilton, Ontario and surrounding areas. No financial compensation was offered to ensure that the recruited participants found the prospect of dating highly motivating in itself, but the cost of transportation was reimbursed. Participants were blind to the hypotheses of the study, and signed informed consent was obtained from each participant in accordance with the Declaration of Helsinki (1991). The McMaster University Research Ethics Board approved all procedures.

### Stimuli

For each date of each session, a different music excerpt was selected as background music. Each excerpt was edited to be 4 min long by truncating the excerpt or looping back to the beginning. Excerpts were presented over a high-quality Meyer Sound 6 channel PA system (Left/Right Main Speakers, Meyer UPJ, Left/Right Front Fill, Meyer UP4, Left/Right Subwoofer, Meyer 500-HP) with intensity around 56 to 64 dB(A), over a noise floor of 10 dB(C) equalized by ReplayGain algorithm in Audacity (2.1.1).

Fifteen excerpts were selected for the experiment out of 43 possible songs. The initial 43 songs were selected in part from the list reported in Janata *et al.* (2012). However, because these songs had highly correlated groove, enjoyment and familiarity ratings, and our goal was to obtain representation from high- and low-groove songs while maintaining approximately similar levels of familiarity and enjoyment, we selected a number of additional songs to be rated. Nineteen raters (aged 20.6 ± 2.3 years, 3 men and 16 women) were recruited to rate the songs. These participants, who did not participate in the speed dating experiment, were students at McMaster University and were blind to the purpose of this study. Using ten-point Likert item, they rated groove (‘how much the music makes you want to move’; Janata *et al.*, 2012), familiarity, and enjoyment levels for a 1-min clip of each of the 43 excerpts. Higher ratings indicated higher groove, familiarity or enjoyment, respectively. The ratings of the 19 raters were moderately consistent with each other (Pearson’s *r*: 0.425 ± 0.066, range 0.259–0.508), but one additional rater’s ratings were excluded due to high inconsistency with the others (Pearson’s *r* = 0.000).

Among the selected 15 excerpts (Table S1), 8 were classified as high-groove (range 6.16–8.11) and 7 as low-groove (range 3.63–5.68) excerpts. A two-sample *t*-test confirmed that the groove ratings between these two sets were statistically different (*t*(13) = 6.72, *P* < 0.001, Cohen’s *d* = 3.73). In contrast, neither enjoyment (*t*(13) = 0.06, *P* = 0.951, Cohen’s *d* = 0.03) nor familiarity (*t*(13) = −0.97, *P* = 0.351, Cohen’s *d* = −0.54) ratings were significantly different between the two levels of groove.

### Procedure and apparatus

The data were collected in the McMaster Large Interactive Virtual Environment laboratory (LIVELab; LIVELab.mcmaster.ca). Fifteen round tables were placed in the LIVELab in three rows. Two chairs were placed face to face at each table, ~1 m apart. Tables were separated by at least 3 m.

Two speed dating sessions were hosted across two evenings, and each participant was allowed to participate in one session only. Face-to-face contact between potential dates prior to the speed dating was minimized by asking men and women to arrive at separate entrances to the lab. Each date was 4 min long, and the participants were instructed to have conversations and interactions only with the dating partner seated at the same table. Immediately following each date, each participant privately completed a short questionnaire (see below) about his/her dating partner on a clipboard. After completing the questionnaire, the men moved to the next table for the next date. Because of 1 to 2 no-show participants per session, when a participant had no dating partner at a table, he/she rested for the duration of that date. There were 15 dates (including rest) per session. Every participant experienced one date with each member of the opposite gender in the same session. The entire session took ~2 h. The experimenter explained the procedure and the questionnaire prior to the speed dating, indicated when each date started and ended, as well as when participants should fill out their questionnaires.

A 4 min music excerpt was played during each date. The excerpts alternated between high- and low-groove, and the order was the same for both sessions (Table S1). The loudness level (see Stimuli section for details) did not interfere with the conversations between dating partners but the music was still audible. Participants were not instructed to pay attention to or to respond to the background music. The presence of background music simulates typical real-world dating environments, such as cafés and restaurants.

An optical motion capture system (24 Oqus 500+ cameras, and 2 Oqus 700+ cameras; Qualisys) recorded participants’ head movements at 104 Hz. Two retroreflective markers (10 mm) were placed on a rigid headband worn by the participants, forming a stable base for the markers. Markers on the headband were placed on the central left and right of the head. One additional marker was placed in the middle of each table as a reference.

### Questionnaire

The questionnaire for each date included (i) MeetingAgain: ‘Would you like to see this person again for a second date?’ (yes/no options), (ii) ‘How interested would you be in seeing this person again for a second date?’, (iii) short-term relationship: ‘How interested would you be in this person as a short-term partner for a brief affair or a one-night stand?’ (iv) long-term relationship: ‘How interested would you be in this person as a long-term partner for a committed, exclusive relationship?’ and (v) attractiveness: ‘Physically, how attractive do you think this person is?’. The answers to questions 2–5 used nine-point Likert item, with higher values indicating greater interest or attractiveness. If both participants in the same date answered ‘yes’ to question 1, the experimenters exchanged their contact information within 24 h of completing the experiment.

Note that question 2 was the nine-point Likert item version of question 1, and the goal was to obtain a more fine-grained response than in question 1. However, 30 out of 54 participants had inconsistent responses across these two questions (i.e., same or higher response on question 2 for partners they rated in question 1 as ‘No, would not want to see this person for a second date’ than for partners rated as ‘Yes, would like to see this person for a second date’). Given that the only difference between questions 1 and 2 was the number of available response points, we suspected that this discrepancy was simply because participants were inconsistent in using the scales across the experiment. Because participants’ responses to question 1 had real-life implications—participants were aware that their responses to question 1 would be used to determine whether they ‘matched’ with mutually interested date partners—participants likely treated question 1 with more importance and it had greater ecological validity than question 2. Therefore, we used question 1 in the primary analyses, but also reported the results of question 2 in Table S5, Supplementary Materials.

### Motion capture data preprocessing

The preprocessing steps followed our previous studies (Chang *et al.*, 2017, 2019). Motion capture data were exported from Qualisys Track Manager (2.16) to MATLAB 2015b for analyses. For each date, the 4 min recorded motion trajectories of the two markers on each participant’s head were gap-filled (spline interpolation). On average, gaps were only 0.18 s long in each 4-min recorded trajectory; only one trajectory had a gap longer than 1 s and this trajectory was excluded from further analysis. The recordings were further down-sampled to 8 Hz (GC prefers a low model order for capturing a given physical time length; Barnett and Seth, 2014), each participant’s two markers were spatially averaged and projected to the anterior-posterior body orientation (referencing to the marker on the table and collapsing altitude), and then z-normalized (to exclude individual differences in movement magnitude) to produce one body sway time series for each participant on each date. We focused on the anterior-posterior body sway orientation, because our previous studies suggested it reflects interpersonal coordination (Chang *et al.*, 2017, 2019).

The multivariate GC toolbox (Barnett and Seth, 2014) was used to estimate the predictive magnitude of a participant’s body sway on his/her partner’s body sway using GC. GC is a statistical estimation, based on vector autoregressive models, of the magnitude of how much one time series is predicted by the history of another time series, taking into account how much it is predicted by its own previous history, in the form of a log-likelihood ratio. The larger the value of GC, the better the prediction and the more information is said to be flowing from one time series to another. First, the toolbox confirmed that each time series passed the stationary assumption for GC analysis, with the spectral radius < 1. Second, an optimal model order (the number of preceding samples included) was estimated by the Akaike information criterion for each date. The optimal model order is a balance between maximizing goodness of fit and minimizing the number of coefficients (length of the time series) being estimated. Finally, in order to compare GC across dates, we fixed the model order at 18 (2.25 s) for all dates, which represented the 95th percentile of the largest optimal model orders across all dates of both sessions. Note that GC is a bidirectional measure, thus the GC body sway coupling of one participant toward his/her partner was not necessarily equal to the coupling in the opposite direction.

To investigate the degree of similarity between partners’ body sways, the cross-correlation was calculated. Cross-correlation quantifies similarity between two time series as a function of a shifting time step between the time series. To empirically compare GC and cross-correlation, we performed cross-correlation analyses on the same preprocessed data to which we had applied GC. For each date, we picked the maximum cross-correlation coefficient (CC) as the index of maximum similarity between partners’ body sways, with lags limited to the GC model order (18 samples), that is, limited to lags between zero and preceding one’s partner’s body sway by 2.25 s. Therefore, the CC body sway coupling of one participant toward his/her partner was not necessarily identical to the counterpart (unless the maximum correlation occurred at lag-0 for both partners).

These two measures provide critically different insights. First, similarity is not the same as predictability. For example, if two time series were identical sinusoidal oscillators, their CC coupling would be perfect, but there would be no predictive coupling by GC, as the second time series cannot add any predictive information that is not already contained in the first time series. Second, CC does not imply directionality, just correlation, whereas GC quantifies directional predictive coupling strengths, such that it is possible for one time series to predict a second time series but at the same time that the reverse is not true (see Dean and Dunsmuir, 2016, for more details).

### Statistical analyses

We used linear mixed-effect models (LMEMs) to investigate how body sway indexes (GC and CC) predict different kinds of romantic interest, while accounting for participants’ individual differences. LMEM is an extension of a linear regression model. Our models assessed the predictors of interest (i.e. fixed effects), while considering variances across participants and dyads (i.e. random effects). Attractiveness was included in each model as a control variable.

Model fitting was implemented using the ‘lme4’ package (1.1–21) (Bates *et al.*, 2015) in R (3.6.1) (R Core Team, 2019). The lmer() function was used when the predicted variable was continuous (interest in short-term relationship or long-term relationship), and the standardized β coefficients were reported as the estimates. The glmer() function was used to perform a logistic generalized LMEM when the predicted variable was binary (MeetingAgain), and the odds ratios were reported as the estimates. The maximum number of iterations was set at 100 000, and the derivative calculation was turned off to facilitate faster processing speed. The significance of the fixed effects was determined with type-II Wald tests using the Anova() function in the ‘car’ package (3.0–3) (Fox and Weisberg, 2019) in R. The odds ratios and standardized β coefficients were calculated by plot_model() function in the ‘sjPlot’ package (2.6.3) (Lüdecke, 2019). If a participant failed to respond to a question for a dating partner, all the other responses for the same dating partner were excluded from analyses for that date. One participant’s data were excluded because she left all MeetingAgain responses blank. In total, for each LMEM, there were 54 clusters (usable participants) and 719 observations.

We did not include the interaction term between gender and GC or CC in the LMEMs, because we did not have any specific predictions about how gender would interact with body sway, and adding this factor and its interactions with GC and CC into the model would greatly increase the number of parameters that would need to be estimated and thus decrease the precision of the model. Nevertheless, gender is known to have important effects on romantic interest (e.g. Fletcher *et al.*, 2014), so we used separate LMEMs for each gender in *post-hoc* analyses (see Supplemental Material).

We treated our Likert data as an ordinal approximation of an interval variable and analyzed it with linear models. Previous studies suggest that it is appropriate of treat Likert data (especially with five or more points) as interval data because (i) empirical and simulation studies have demonstrated that linear and non-linear (ordinal, ranked) approaches of analyzing Likert data show highly similar results, (ii) linear tests are robust to the violation of statistical assumptions to a great degree and (iii) linear approaches have higher statistical power than non-linear alternatives (Johnson and Creech, 1983; Liu *et al*., 2017; Norman, 2010; Sullivan & Artino, 2013; Zumbo and Zimmerman, 1993).

Every statistical test was performed two-tailed. We set α = 0.05, and Bonferroni-adjustment of α = 0.05/3 ≅ 0.017 was used for each family of three tests (MeetingAgain, short-term relationship and long-term relationship) as a conservative control for type I error. The effect size (semipartial *R*^2^) of each variable of lmer() was calculated by Kenward Roger approach with function r2beta() of the ‘r2glmm’ package (0.1.2) in R (Jaeger, Edwards, Das, & Sen, 2017).

## Results

The datasets analyzed during the current study are available in the Open Science Framework repository, https://osf.io/9fr2a/. The raw time series can be provided on a reasonable request and agreement.

### Directional body sway coupling predicts interest in a long-term relationship

First, we used both GC (directional coupling) and CC (similarity coupling) body sway indexes along with attractiveness (control variable), without interactions, to predict interest in MeetingAgain (yes/no) (Table 1). Given the nested nature of the current experiment, participants (intercept and slope) and dyads (intercept) were included as random effects. The logistic generalized LMEM showed a strong effect of attractiveness, such that participants were more interested in having a second date with dating partners who were perceived to be more physically attractive. There was a trend for GC to positively predict MeetingAgain, but it did not reach the significance threshold (0.05/3). There was no effect of CC on MeetingAgain.

**Table 1:** Predicting romantic interests with attractiveness and body sway coupling indexes (GC and CC)

Variable	Odds ratio/standardized β coefficient	SE	χ^2^(1)	*P*-value	semipartial *R*^2^
MeetingAgain (logistic generalized LMEM)					
Attractiveness	6.66	0.65	104.96	<0.001	not applicable (N/A)
GC (directional)	0.68	0.29	5.38	0.020	N/A
CC (similarity)	-0.34	0.31	1.17	0.280	N/A
Short-term relationship (LMEM)					
Attractiveness	0.45	0.04	124.63	<0.001	0.69
GC (directional)	0.02	0.02	1.42	0.234	0.03
CC (similarity)	0.01	0.02	0.63	0.429	0.01
Long-term relationship (LMEM)					
Attractiveness	0.63	0.04	238.54	<0.001	0.81
GC (directional)	0.06	0.02	6.43	0.011	0.12
CC (similarity)	-0.04	0.02	3.22	0.073	0.06

Note: The odds ratios are reported for the variables in the logistic generalized LMEM (MeetingAgain), and the standardized β coefficients are reported for the variables in the LMEMs (short-term and Long-term relationships). The Bonferroni-controlled statistical significance threshold is 0.05/3 ≅ 0.017. The semipartial *R*^2^s are only available to the variables in LMEMs.

Second, we used LMEM with the same fixed and random effects without interactions to predict interest in a short-term relationship (Table 1). The LMEM again showed a strong effect of attractiveness, such that participants were more interested in having a short-term relationship with dating partners who they perceived to be more physically attractive. There was no significant effect of GC or CC on short-term relationship.

Finally, the same LMEM as above was used to predict interest in a long-term relationship (Table 1). Once again, the LMEM demonstrated that participants were more interested in having a long-term relationship with dating partners who were perceived to be more physically attractive. After accounting for attractiveness, the GC of body sway was associated with interest in a long-term relationship with medium effect size, such that a participant’s interest in a long-term relationship was higher if their body sway directionally predicted their partner’s body sway. However, the effect of CC on interest in a long-term relationship did not reach the significance threshold (0.05/3).

*Post-hoc* analyses using separate LMEMs for each gender are reported in Supplemental Material and Table S2-S4), but none of the effects reached the corrected statistical threshold (0.05/6).

### Effects of groove and gender on romantic interest

**Fig. 1:**
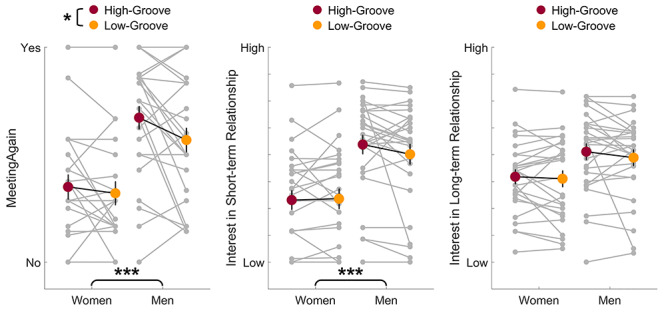
The effects of participants’ gender and high-/low-groove level of background music on romantic interest. Each grey dot represents one participant’s data, and each grey line connects the same participant’s data under different conditions. The red and yellow dots represent the group mean under high-groove or low-groove condition, respectively. Error bars represent the mean ± one standard error. *P < 0.05/3; ***P < 0.001.

For each measure of romantic interest (MeetingAgain, short-term relationship, long-term relationship), we took each participant’s average romantic interest ratings and conducted a two-way mixed-effect ANOVA with between-subject factor gender and within-subject factor groove (Figure 1), with corrected statistical thresholds (0.05/3). Participants had higher interest in MeetingAgain when the background music of their date was high-groove than low-groove (*F*(1,52) = 6.37, *P* = 0.015, *η^2^* = 0.11), and men were more interested in general in meeting their dating partner again than women (*F*(1,52) = 15.76, *P* < 0.001, *η^2^* = 0.23). No significant interaction effect was observed (*F*(1,52) = 1.96, *P* = 0.168, *η^2^* = 0.04). Men were more interested than women in pursuing short-term relationships with their dating partner, (*F*(1,52) = 14.79, *P* < 0.001, *η^2^* = 0.22), but there was no significant effect of groove (*F*(1,52) = 1.20, *P* = 0.279, *η^2^* = 0.02) or interaction effect (*F*(1,52) = 2.11, *P* = 0.152, *η^2^* = 0.04). For interest in a long-term relationship, there were no significant effects of gender (*F*(1,52) = 4.99, *P* = 0.030, *η^2^* = 0.09), groove (*F*(1,52) = 1.47, *P* = 0.231, *η^2^* = 0.03), or interaction effect (*F*(1,52) = 0.35, *P* = 0.559, *η^2^* = 0.01). In general, we replicated previous findings that men generally are more interested in a wider range of partners than women, and that physical attractiveness affects short-term more than long-term romantic interest (e.g. Fletcher *et al.*, 2014).

### No evidence that groovy music enhances body sway coupling

In separate analyses, we observed that both body sway GC coupling and groovy music were significantly associated with romantic interest. One possible explanation is that groovy music potentially encouraged more movement in the participants, creating more opportunities for body sway coupling, leading to more romantic interest. We used LMEMs to analyze the fixed effects of groove, gender and their interaction on body sway coupling, with participants (intercept and slope) and dyads (intercept) as random effects. However, we found no evidence that groovy music or gender associates with body sway coupling (Table 2).

**Table 2:** Predicting body sway coupling index (GC or CC) with gender and high-/low-groove level of background music

Variable	Standardized β coefficient	SE	χ^2^(1)	*P*-value
GC				
Gender (male)	−0.03	0.07	0.42	0.519
Groove (low-groove)	0.01	0.05	0.00	0.950
Interaction	−0.03	0.06	0.19	0.659
CC				
Gender (male)	−0.01	0.07	0.02	0.895
Groove (low-groove)	−0.02	0.06	0.21	0.646
Interaction	0.00	0.06	0.00	0.989

Note: The semipartial *R*^2^ is not reported here due to convergence failure, which might be the results of near zero effects.

## Discussion

We used speed dating as a research paradigm to investigate the effects of body sway and groovy music on different aspects of romantic interest. This study was primarily concerned with the novel questions of whether body sway coupling predicts initial interest in romantic relationships and, if so, whether the nature of that movement is based on directional coupling (GC) or similarity coupling (CC). We found that directional body sway coupling predicted interest in long-term, but not short-term relationships. Specifically, participants had higher interest in a long-term relationship if their body sway dynamics better predicted their partner’s body sway, even when controlling for perceived physical attractiveness. In addition, the type of background music influenced participants’ choices. Specifically, participants were more interested in meeting their dating partners again when the background music was high-groove than when it was low-groove.

Our study established a novel behavioral index of long-term romantic interest. Interestingly, directional body sway coupling predicts interest in long-term but not short-term relationships. This is consistent with previous literature showing interest in short-term relationships is primarily associated with attractiveness, while interest in long-term relationships is associated with similar and socially appealing personality characteristics, like intelligence, honesty, and warmth (e.g. Regan *et al.*, 2000). We propose that directional body sway coupling reflects the quality of inter-partner communication, which reveals the degree of compatible personality characteristics. This is consistent with our music ensemble studies, in which directional body sway coupling reflected directional interpersonal communication and predicted the quality of ensemble musical performances (Chang *et al.*, 2017, 2019).

We did not find that similarity of body sway coupling predicted any type of romantic interest. If anything, there was a trend for a negative effect in that the participants had less long-term romantic interest if their body sway was more similar to that of their partner. While this appears at odds with previous studies demonstrating that manipulated interpersonally synchronized movement promotes interpersonal affiliation (e.g. Hove and Risen, 2009; Wiltermuth and Heath, 2009), a major difference is that the current study did not directly manipulate movement synchrony. Synchronized movements might promote romantic interest, but only if the movements are externally manipulated, as through, for example, explicit instructions, behavior of a confederate or in response to the beat of music. Another possibility is that synchrony has different effects on romantic interest and interpersonal affiliation. Note, of course, that these *post-hoc* speculations are based on null statistical differences and further investigations are needed.

Numerous social psychology studies show that music preferences are often associated with attraction, closeness and relationship satisfaction (e.g. Boer *et al.*, 2011; Rentfrow, 2012; Ferguson *et al.*, 2016), but how background music directly influences people’s romantic interest during face-to-face interactions is largely unexplored. Our findings show that groovy background music promotes romantic interest during speed dating, warranting future investigations on this topic. However, we found no evidence that this was related to enhanced body sway coupling during high-groove dates, which was not consistent with our hypothesis. Previous findings show that groovy music promotes movement entrainment to the music (e.g. Janata *et al.*, 2012; Ross *et al.*, 2016), and thus might be expected to result in increased similarity of body movement between partners. This effect might be attenuated, however, if participants are actively engaged in another task like having a conversation, as in the present case. An alternative possible explanation for the relation between groovy music and romantic interest is that groove-enhanced arousal levels might be misattributed to romantic interest (Marin *et al.*, 2017; Bowling *et al.*, 2018). Again, this possibility would need to be explored in future research.

A strength of the approach taken here is that it used an ecologically valid real speed dating situation to examine non-verbal aspects of initial romantic interest and the potential role of background music in its formation. As a consequence, factors such as unique effects of particular dyads were difficult to control. We did include dyad as a random factor in our models, but we were not able to delve more deeply into possible differences between dyads. Nonetheless, we can conclude that romantic interest is associated with body sway directional predictive coupling (GC), and that groovy music can promote romantic interest. These findings provide novel perspectives and approaches to investigate social bonding in dynamic real-world settings.

## Supplementary Material

nsaa093_Supp
