# Supplementary material for: Body sway predicts romantic interest in speed dating
Source: Soc Cogn Affect Neurosci. 2020 Jul 18;16(1-2):185–92. doi: 10.1093/scan/nsaa093 (PMC7812630; doi:10.1093/scan/nsaa093)
Supplement: nsaa093_Supp [file nsaa093_supp.zip › scan-20-024-File004_nsaa093.pdf]

Supplemental Material

Body sway predicts romantic interest in speed dating

Background music selection

The ratings from the current study for the pieces used, as well as the ratings from Janata et al. (2012) where available, are shown in Table S1.

Table S1. Background music pieces and their ratings

			Current study (scale: 1-10)			Janata et al. (2012)
**Order**	**Song name**	**Artist**	**Groove**	**Enjoyment**	**Familiarity**	**Groove (scale: 0-127)**
1	Flurries	Soulive	6.26	5.32	5.11	87.8
2	America	Simon & Garfunkel	4.11	5.79	5.74	N/A
3	Flash Light	Parliament	7.84	7.16	6.16	105.1
4	Comfortably Numb	Pink Floyd	4.26	5.79	5.89	42.3
5	Lady Marmalade	LaBelle	7.79	7.53	8.42	102.5
6	Daughters	John Mayer	5.05	7.11	8.11	N/A
7	Superstition	Stevie Wonder	8.05	7.26	8.26	108.7
8	Good Riddance (Time of Your Life)	Green Day	4.95	7.53	9.05	N/A
9	Mama Cita	Funk Squad	7.63	6.84	6.05	101.6
10	Hey There Delilah	Plain White T's	5.68	8.37	9.53	N/A
11	Music	Leela James	6.16	6.11	5.79	101.1
12	I Will Follow You Into the Dark	Death Cab for Cutie	3.89	6.32	7.58	N/A
13	Naughty Girl	Beyoncé	8.11	7.53	8.95	87.0
14	Space Oddity	David Bowie	3.63	5.05	6.32	38.7
15	Outa-Space	Billy Preston	6.42	5.05	4.53	90.9

N/A: not applicable

No gender differences on body sway coupling predicting romantic interest

While we did not have specific predictions about how gender and body sway coupling would interact, gender has an important role in many dating contexts, so we investigated potential gender differences in a secondary exploratory analysis. Because including gender as a binary predictor weakened the robustness of the LMEM, we split the data based on gender and

performed separate post-hoc LMEMs. There were 27 clusters and 358 observations of the LMEMs for men, and 27 clusters and 361 observations of the LMEMs for women.

We used fixed effects GC and CC body sway indexes along with Attractiveness (without interactions) to predict romantic interest (MeetingAgain, Short-term Relationship, Long-term Relationship) with participants (intercept and slope) and dyads (intercept) as random effects. The results are summarized in Table S2-4. Overall, the results showed a strong positive association between Attractiveness and all aspects of romantic interests measured here in both men and women. However, we did not find evidence for differences in the way that men and women evaluated different kinds of romantic attraction based on attractiveness, GC coupling, or CC coupling with their dating partners. There were no effects for women. For men, there was a numerical trend of the effect of GC on interest in a Long-term Relationship, but this effect did not reach the corrected statistical threshold (0.05/6). Likely, there was not enough power to detect such effects, so these negative findings should be treated with caution.

Table S2. Logistic generalized LMEM on MeetingAgain

Variable	Odds ratio	SE	$\chi^2(1)$	p-value
Men				
Attractiveness	6.52	0.84	60.28	< 0.001
GC	0.87	0.47	3.48	0.062
CC	-0.50	0.42	1.46	0.227
Women				
Attractiveness	6.87	1.02	45.12	< 0.001
GC	1.11	0.52	4.52	0.034
CC	-0.05	0.62	0.01	0.938

Table S3. LMEM on Short-term Relationship

Variable	Standardized beta coefficient	SE	$\chi^2(1)$	p-value	semipartial R^2
Men					
Attractiveness	0.52	0.06	84.82	< 0.001	0.75
GC	0.02	0.03	0.63	0.426	0.03
CC	0.00	0.03	0.02	0.877	< 0.01
Women					
Attractiveness	0.42	0.06	45.55	< 0.001	0.62
GC	0.02	0.03	0.48	0.487	0.02
CC	0.03	0.03	1.26	0.261	0.05

Table S4. LMEM on Long-term Relationship

Variables	Standardized beta coefficient	SE	$\chi^2(1)$	p-value	semipartial R^2
Men					
Attractiveness	0.66	0.07	91.85	< 0.001	0.77
GC	0.08	0.03	5.80	0.016	0.19
CC	-0.03	0.03	0.70	0.404	0.03
Women					
Attractiveness	0.62	0.05	177.54	< 0.001	0.85
GC	0.05	0.03	1.71	0.191	0.06
CC	-0.06	0.04	2.33	0.127	0.08

Table S5. LMEM on Q2 (interest in seeing this partner again, 9-point Likert data)

Variables	Standardized beta coefficient	SE	$\chi^2(1)$	p-value	semipartial R^2
Attractiveness	0.70	0.04	352.16	<0.001	0.85
GC	0.04	0.02	2.42	0.120	0.05
CC	-0.02	0.02	0.54	0.461	0.01

Body sway coupling of partner-predicting-self or partner-preceding-self has no effect on romantic interest

In all the preceding analyses, we focused on whether romantic interests can be predicted by the body sway coupling of self-predicting-partner (GC, directional) and/or self-preceding-

partner (CC, similarity). An alternative approach is to reverse the predicting and preceding relationships in the LMEMs; in other words, whether romantic interest is associated with the partner-predicting-self GC and/or partner-preceding-self CC body sway couplings. We conducted LMEMs on romantic interest (MeetingAgain, Short-term Relationship, Long-term Relationship) with fixed effects partner-predicting-self GC and partner-preceding-self CC body sway (along with Attractiveness, without interactions) and participants (intercept and slope) and dyads (intercept) as random effects. However, our analyses did not show any significant body sway coupling effects under the corrected statistical threshold (0.05/3) (Table S6). The only marginal effect was that the GC coupling negatively associated with short-term romantic interest. Nevertheless, again, this effect did not reach the corrected statistical threshold, and future studies are needed to investigate this trend.

Table S6. Predicting romantic interests with Attractiveness and body sway coupling indexes of partner-predicting-self (GC, directional) and partner-preceding-self (CC, similarity).

Variable	Odds ratio/ Standardized beta coefficient	SE	$\chi^2(1)$	p-value	semipartial R^2
MeetingAgain					
Attractiveness	6.47	0.62	107.88	<0.001	N/A
GC	0.02	0.28	0.01	0.939	N/A
CC	-0.18	0.32	0.32	0.574	N/A
Short-term Relationship					
Attractiveness	0.45	0.04	126.90	<0.001	0.69
GC	-0.04	0.02	5.58	0.018	0.13
CC	0.03	0.02	2.76	0.097	0.06
Long-term Relationship					
Attractiveness	0.62	0.04	233.58	<0.001	0.80
GC	-0.04	0.02	2.84	0.092	0.07
CC	-0.02	0.02	0.83	0.361	0.02

Note: The corrected statistical threshold is $0.05/3 \cong 0.017$.
